# Free-living amoebae and their associated bacteria in Austrian cooling towers: a 1-year routine screening

**DOI:** 10.1007/s00436-016-5097-z

**Published:** 2016-05-14

**Authors:** Ute Scheikl, Han-Fei Tsao, Matthias Horn, Alexander Indra, Julia Walochnik

**Affiliations:** 1 Institute of Specific Prophylaxis and Tropical Medicine, Medical University of Vienna, Vienna, Austria; 2 Department of Microbiology and Ecosystem Science, Division of Microbial Ecology, University of Vienna, Vienna, Austria; 3 Department of Mycobacteriology and Clinical Molecular Biology, AGES, Vienna, Austria

**Keywords:** Free-living amoebae, Screening, Real-time PCR, *Acanthamoeba*, *Legionella*, Cooling towers

## Abstract

Free-living amoebae (FLA) are widely spread in the environment and known to cause rare but often serious infections. Besides this, FLA may serve as vehicles for bacterial pathogens. In particular, *Legionella pneumophila* is known to replicate within FLA thereby also gaining enhanced infectivity. Cooling towers have been the source of outbreaks of Legionnaires’ disease in the past and are thus usually screened for legionellae on a routine basis, not considering, however, FLA and their vehicle function. The aim of this study was to incorporate a screening system for host amoebae into a *Legionella* routine screening. A new real-time PCR-based screening system for various groups of FLA was established. Three cooling towers were screened every 2 weeks over the period of 1 year for FLA and *Legionella* spp., by culture and molecular methods in parallel. Altogether, 83.3 % of the cooling tower samples were positive for FLA, *Acanthamoeba* being the dominating genus. Interestingly, 69.7 % of the cooling tower samples were not suitable for the standard *Legionella* screening due to their high organic burden. In the remaining samples, positivity for *Legionella* spp. was 25 % by culture, but overall positivity was 50 % by molecular methods. Several amoebal isolates revealed intracellular bacteria.

## Introduction

Free-living amoebae (FLA) have a worldwide distribution and are found in various natural habitats like soil, freshwater and seawater (Smirnov and Brown 2004; Berk et al. 2006; Geisen et al. 2014). Additionally, they can colonize engineered water facilities, including water treatment plants, air conditioning units, plumbing systems, drinking water networks and cooling towers (Delafont et al. 2013; Retana-Moreira et al. 2014; Canals et al. 2015). In all of these habitats, FLA play an important role as vehicles of replication and dispersal for bacteria (Cirillo et al. 1997; La Scola and Raoult 2001; Winiecka-Krusnell and Linder 2001; Greub and Raoult 2004; Siddiqui and Khan 2012). The extremely resilient genus *Acanthamoeba* is a particularly well-suited host for a long list of bacteria, including *Legionella pneumophila*, the causative agent of Legionnaires’ disease, a severe pneumonia (Rowbotham 1980). In the amoebae, the legionellae are protected from changes in pH and temperature and from disinfection (Wadowsky et al. 1985; Ohno et al. 2003; Hwang et al. 2006; Dupuy et al. 2011; Cervero-Aragó et al. 2014). Moreover, passage through amoebae seems to enhance their virulence and to resuscitate viable but non-culturable (VBNC) legionellae (Cirillo et al. 1999; Steinert et al. 1997; García et al. 2007). Under environmental stress, legionellae can enter the VBNC state, a physiological dormant state, in which they cannot be detected by standard cultivation techniques (Hussong et al. 1987; Robertson et al. 2014).

Man-made habitats like open cooling towers can disseminate legionellae via aerosols (Walser et al. 2014). These aerosols can be distributed over long distances up to 6 km (Addiss et al. 1989). Cooling towers of large public buildings pose a particular risk and have been reported as sources of community-acquired and nosocomial outbreaks of Legionnaires’ disease (Nguyen et al. 2006; Hugosson et al. 2007; Sala Ferré et al. 2009; Freudenmann et al. 2011; Buse et al. 2012). An example for such an incidence in Austria was reported in 2007 (Wewalka 2013), when the cooling towers of a hospital were the source of a *Legionella* outbreak with 16 cases of legionellosis, including three lethal ones. The cases occurred not only in the hospital itself but also in its surroundings. Until now, legislation in Austria does neither require the registration of wet cooling systems nor are regular microbiological monitoring or standard disinfection mandatory. Most public cooling towers are, however, screened on a routine basis by the respective operating company. Thus, a project was initiated to incorporate a screening system for host amoebae into a routine screening program for legionellae. A particular aim of this study was to synchronously assess the diversity of FLA relevant as bacterial hosts and to also investigate all amoebal isolates for intracellular bacteria. Thus, a rapid and reliable screening system for the detection and synchronous differentiation of the host amoebae was established.

## Materials and methods

### Sample collection and processing

The study included three open cooling towers that are under permanent observation for legionellae and under regular albeit not standardized disinfection. The routine screening in operation also includes *Pseudomonas aeruginosa* and total bacteria. Two of the cooling towers were hospital cooling towers, belonging to two different hospitals, one in the centre of Vienna (CT-Hospital 1) and the other one in the periphery of Vienna (CT-Hospital 2). Cooling towers of Hospital 1 had been the source of the *Legionella* outbreak in 2007 and since then had been dismantled and renewed. The third cooling tower (CT-Company) was from a complex of company buildings, located in the same district as CT-Hospital 1 and comprising several large office buildings with bureaus, shops, restaurants, a kindergarten and also an outpatient clinic. Moreover, 12 tap waters (Tap) from various sites were sampled over the same period of time for comparison, including one sample from a previously *Legionella*-contaminated shower head in another hospital in Vienna. During the study period of 1 year, water samples were obtained every 14 days, corresponding to the recommendations for routine *Legionella* screening. CT-Company was not sampled between November 2013 and March 2014 as the system was not in operation. Currently, there is no standardized disinfection protocol for cooling towers in Austria. The cooling towers included in this study are disinfected over a dosimeter with chlorine and bromine-based oxidative biocides. During the study, CT-Hospital 1 was disinfected three times a week (OptiDOS 39D®, OptiDOS DSB®), CT-Hospital 2 every 18 h (Waterdos CLD 08®) and CT-Company every 4 days (Waterdos CIT48®). As we have shown in a previous study that amoebae re-colonize waters rapidly after disinfection (Scheikl et al. 2014) and to have stringent conditions for the frequency of occurrence of amoebae, samples were always obtained within 24 h after disinfection. Water temperatures from all sites were recorded throughout the study.

Sampling procedure and evaluation strictly followed the new requirements for evaporative re-cooling plants (ÖNORM B 5020:^2013^ Austrian Standards Institute 2013). From each sampling site, 3 L of water was collected in sterile plastic bottles, stored at 4 °C and processed within 48 h. For direct DNA isolation from water samples, 2 L was filtered and DNA was extracted from cellulose nitrate filters using the MO BIO PowerWater® DNA Isolation Kit (MO BIO Laboratories Inc., Carlsbad, CA). The isolated DNA was stored at −20 °C until use for molecular analyses.

### Screening for FLA

For the detection of *Acanthamoeba* spp., a real-time PCR assay (Qvarnstrom et al. 2006) was adapted, using the primers AcantF900 5′-CCCAGATCGTTTACCGTGAA-3′, AcantR1100 5′-TAAATATTAATGCCCCCAACTATCC-3′ and the Cy5-labelled probe AcantP1000 5′-Cy5-CTGCCACCGAATACATTAGCATGG-BHQ3-3′ and amplifying fragments of 170 to 230 bp, depending on the genotype. For design of the primers and probe for the detection of Vahlkampfiidae, particularly *Naegleria* spp., we retrieved partial or full length 18S rDNA sequences from GenBank (NCBI, National Center for Biotechnology Information) and included them in multiple sequence alignments. Sequences of *Naegleria jamiesoni*, *Naegleria andersoni*, *Naegleria clarki*, *Naegleria andersoni*, *Naegleria fultoni*, *Naegleria pagei*, *Naegleria australiensis*, *Naegleria lovaniensis*, *Naegleria fowleri*, several unidentified *Naegleria* spp. strains and additionally, 13 sequences from other vahlkampfiids including *Paravahlkampfia*, *Vahlkampfia*, *Singhamoeba*, *Willaertia* and *Tetramitus* were evaluated and compared for conserved and variable regions resulting in the new primers VahlNaegF 5′-GTATAGTCGCAAGACCGAAAC-3′, VahlNaegR 5′-CAAGACAGATCACTCCACGA-3′ and the Cy5-labelled probe VahlNaegP 5′-Cy5-GAAAGGCACCACCAGGAGTG-BHQ2-3′, amplifying a 190–200-bp fragment of the 18S rDNA. The same procedure was followed for the design of the primers and probe for the detection of *Vermamoeba vermiformis*, namely VermHartF 5′-TAACGATTGGAGGGCAAGTC-3′, VermHartR 5′-ACGCCTGCTTTGAACACTCT-3′ and the HEX-labelled probe VermHartP 5′-HEX- TGGGGAATCAACCGCTAGGA-BHQ1-3′ with an amplicon of approximately 240 bp in length. The specificity of all primers and probes was evaluated with Primer3Plus, BLAST Nucleotide search and multiple alignments with other amoebal genera. Moreover, PCR test runs were performed with several reference strains, to check specificity and sensitivity. The PCRs were duplexed with an Exogenous Internal Positive Control (IPC) to distinguish true target negatives from PCR inhibition (Behets et al. 2007). Real-time PCRs were performed in a final reaction volume of 20 μl, containing 1× TaqMan® Fast Universal PCR Mastermix (Applied Biosystems, USA), forward primer (0.9 μM), reverse primer (0.9 μM), probe (0.25 μM), 1× Exo IPC Mix, 1× Exo IPC, 3 μl DNA and sterile H_2_O (for DNA analysis, Carl Roth, Germany). PCRs with no IPC signal were repeated with tenfold diluted DNA. Real-time PCRs were performed in a Light Cycler® LC 480 Instrument (Roche, Germany) with an initial activation step at 95 °C for 10 min followed by 45 cycles of 95 °C for 15 s and 60 °C for 60 s. Fluorescence was measured at the end of the 60 °C anneal/extend step. Samples with a Ct (threshold cycle) value below 40 were considered to be positive. The cell detection limit for *Acanthamoeba* and Vahlkampfiidae was below one cell whereas the detection limit for *V. vermiformis* was about three cells. Data were analysed with the LightCycler® 480 Software (version 1.5) and calculated using the second-derivate maximum algorithm.

### Reference strains

For the establishment of the real-time PCR assays and as controls, we used amoeba reference strains from our culture collection, namely *Acanthamoeba polyphaga* strain 4CL, genotype T4 (ATCC PRA-107^TM^; Walochnik et al. 2000), *Acanthamoeba castellanii* strain 1BU, genotype T4 (ATCC PRA-105^TM^), *V. vermiformis* strain 1282-2 (isolated from a contact lens case, 2010), *Hartmannella cantabrigiensis* strain Hc (Walochnik et al. 1999), *Naegleria lovaniensis* strain 12N (veterinary faeces sample, 2005) and *Naegleria gruberi* strain 40N (GenBank accession no. AF114439). From each reference strain DNA was extracted from tenfold dilution series (10^5^ cells/ml to 1 cell/ml), so that the highest diluted sample contained less than one amoebal cell per 20 μl reaction mix.

### FLA culture and identification

For isolation of FLA, 250 ml of well-mixed water samples was vacuum-filtered through a cellulose nitrate filter with 0.45 μm pore size (area 12.5 cm^2^, Sartorius, Germany). After filtration, each filter was placed up-side down onto a NN (non-nutrient) agar plate covered with 100 μl of a 48-h-old culture of *Escherichia coli* in brain-heart-infusion-broth (BHI) (Sigma-Aldrich, Vienna, Austria). In order to give the amoebae more opportunities to migrate into the bacterial lawn, the filters were cut into two pieces before placing them onto the plates. The plates were sealed with Parafilm® and stored at room temperature for up to 4 weeks, investigating them daily for amoebal growth by inverted phase contrast microscopy (Nikon TMS). Detected FLA were transferred to fresh *E. coli*-coated NN plates using a sterile inoculation loop. All amoebal isolates were cloned by sub-culturing to generate pure cultures for subsequent DNA isolation. Morphological identification was accomplished by inverted phase contrast and bright field microscopy (Nikon Eclipse E800) using the identification keys of Page (Page 1991) and Smirnov (Smirnov et al. 2011). Trophozoites from clonal cultures were harvested with cotton swabs and re-suspended in 15 ml centrifuge tubes filled with 5 ml 0.9 % sodium chloride (NaCl). The samples were centrifuged for 10 min at 800×*g*, the supernatants were discarded and the pellets were re-suspended in 200 μl 0.9 % NaCl. Total genomic DNA was extracted from the cells using the QIAmp® DNA Mini Kit (QIAGEN, Hilden, Germany). Genotyping of *Acanthamoeba* isolates was performed by amplifying and sequencing a 385–450-bp (depending on the genotype) long fragment of the *Acanthamoeba*-specific amplimer ASA.S1 located in the 18S rRNA gene using the newly designed primer AcF1 5′-TGCCACCGAATACATTAGCAT-3′ and AcR1 5′-ACAAGCTGCTAGGGGAGTCA-3′, modified from the JDP2 primer from Schroeder et al. (2001). PCRs were run with 1, 3 and 6 μl whole cell DNA in a total reaction volume of 50 μl for each sample under the following conditions: 15 min pre-heating at 95 °C, followed by 35 cycles at 95 °C for 1 min, 60 °C for 2 min, 72 °C for 3 min and a final extension for 7 min at 72 °C. An ASA.S1 amplicon clone of a T4 genotype strain was used as a positive control. *Acanthamoeba* genotypes were assessed with the model assumption of a <5 % sequence dissimilarity within one genotype (Gast et al. 1996). DNA extracted from other amoebae isolated by culture was amplified and sequenced using universal eukaryotic primers binding in the 18S rRNA gene, namely the modified primers SSU1 5′-CGACTGGTTGATCCTGCCAGTAG3′ and SSU2 5′-TCCTGATCCTTCTGCAGGTTCAC-3′ (Gast et al. 1994) and P1fw 5′-CAAGTCTGGTGCCAGCAGC-3′, P1rev 5′-GCTGCTGGCACCAGACTTG-3′, P2fw 5′-GATCAGATACCGTCGTAGTC-3′, P2rev 5′-GACTACGACGGTATCTGATC-3′, P3fw 5′-CAGGTCTGTGATGCCCTTAG-3′ and P3rev 5′-CTAAGGGCATCACAGACCTG-3′ (Walochnik et al. 2004). PCR was performed with 1, 3 and 6 μl of whole cell DNA in 50 μl reaction volume running a standard amplification program (35 cycles; 95 °C for 1 min, 52 °C for 2 min, 72 °C for 3 min). Amplified DNA was detected by gel electrophoresis on a 2 % agarose gel and visualized with GelRed™ (BIOTREND, Germany). Gel bands were extracted with the GFX PCR DNA and Gel Band Purification Kit (GE Healthcare, UK) and directly sequenced in both directions with the ABI PRISM® BigDye sequencing kit and an ABI PRISM 310® automated sequencer (PE Applied Biosystems, Germany). All sequences obtained were compared to sequences from GenBank with the NCBI Nucleotide BLAST search and aligned with sequences of highest similarity using ClustalX (Thompson et al. 1997) or CLC Main Workbench (CLC bio, QIAGEN). Multiple alignments were processed with the GeneDoc sequence editor (Nicholas et al. 1997).

### Screening for intracellular bacteria

All isolates were screened for intracellular bacteria. Endosymbionts were demonstrated by FISH (fluorescence in situ hybridization) using the probe EUK516 (5′- ACCAGACTTGCCCTCC -3′), detecting most Eukaryotes, a mix of bacterial probes, namely EUB338 I-III (5′-GCTGCC TCCCGTAGGAGT-3′, 5′-GCAGCCACCCGTAGGTGT-3′, 5′-GCTGCCACCCGTAGGTGT-3′; Amann et al. 1990; Daims et al. 1999) and the negative control probe NONEUB (5′-ACTCCTAC GGGAGGCAGC-3′). Amplification and identification were performed by 16S rRNA gene sequencing using the forward primer 27F (5’-AGAGTTTGATCCTGGCTCAG-3’) and reverse primer 1492R (5’-GGTTACCTTGTTACGACTT-3’) (Lane 1991).

### Routine screening for bacteria

The bacterial screening was performed according to the respective international regulations. The water samples were analysed for *Legionella* spp. CFU/100 ml (colony forming units in 100 ml) after centrifugation and filtration of 100 ml untreated water and after acid treatment, respectively (ISO 11731-2 2004). *Legionella* species were identified by sequencing the mip-gene and *L. pneumophila* was serotyped according to the EWGLI- (European Working Group for Legionella Infections 2011) criteria. If despite acid treatment organic burden prohibited filtration of 100 ml, smaller volumes, i.e. 1–10 ml, were used, as is standard procedure. For comparison, a random set of 28 cooling tower samples were also analysed by amplicon sequencing for the presence of operational taxonomic units (OTUs) classified as members of the genus *Legionella*.

*P. aeruginosa* was evaluated in 100 ml of water (ISO 16266 2008) and total heterotrophic bacteria were counted as CFU in 1 ml at 36 °C (ISO 6222 1999).

### Amplicon sequencing and analysis

Amplicon sequencing was performed as described in Herbold et al. (2015). The V3 and V4 regions of the bacterial 16S rRNA were amplified with barcoded versions of the primers Bakt_341F (CCTACGGGNGGCWGCAG) and Bakt_805R (GACTACHVGGGTATCTAATCC) (Herlemann et al. 2011). Each PCR reaction included 1× DreamTaq Green Buffer (Fermentas, Thermo Fisher Scientific, Vienna, Austria), 2 mM MgCl_2_, 0.2 mM dNTP mix (Fermentas), 0.1 mg ml^−1^ bovine serum albumin, 1 μM of each of the forward and reverse primers, 0.025 U DreamTaq polymerase (Fermentas) and 1 μl of template. The PCR was performed with a cycle ratio of 25:10. Clustering into OTUs was performed as described previously (Herbold et al. 2015). Taxonomic classification was carried out using the mothur classify.seqs function (Schloss et al. 2009) and the Silva 1.19 SSU database as reference (Quast et al. 2013). The bootstrap threshold for the taxonomic assignment was set to 80 %.

### Statistical analysis

The collected data were analysed with IBM SPSS Statistics, version 19 (SPSS Inc., Chicago, USA). Frequency distributions were compared using chi-square tests or Fisher’s exact probability test, as appropriate. Dependent frequencies were compared by McNemar tests. For all analyses, *p* values below 0.05 were considered significant.

## Results

The two hospital cooling towers showed temperatures between 20 and 30 °C throughout the entire year, with mean temperatures of above 25 °C. The company cooling tower showed temperatures below 20 °C at the beginning of the operating time and this cooling tower was not in use during the winter (November to March). The tap water samples had temperatures between 4 and 12 °C.

### Screening for FLA relevant as hosts for bacteria

Altogether, 83.3 % (55/66) of the cooling tower samples and 50 % (6/12) of the tap water samples were positive for FLA (Table 1). The shower head sample was negative in all three real-time PCR tests (not shown). *Acanthamoeba* was the most prevalent genus, detected in 71.2 % of all cooling tower samples and in 50 % of the tap water samples (Table 1). In tap water, *Acanthamoeba* was the only genus of FLA found, while cooling waters also had a high frequency of occurrence of vahlkampfiids (57.6 % positive samples). In 45.5 % of all cooling tower samples, *Acanthamoeba* co-occurred with vahlkampfiids, with the highest numbers of samples being positive for both taxa in CT-Hospital 1 (65.4 %). In comparison, CT-Hospital 2 showed significantly higher frequencies of *Acanthamoeba* than of Vahlkampfiidae (*p* = 0.01). The genus *Vermamoeba* always co-occurred with vahlkampfiids (7.6 %) and in 4.5 % of the samples all three groups of FLA were detected simultaneously.

**Table 1:** Positivity rates of FLA in cooling towers and tap waters, evaluated by real-time PCR

FLA		Cooling tower Hospital 1	Cooling tower Hospital 2	Cooling tower Company	Tap water	Total
*Acanthamoeba*	%^a^ Number^b^	69.2 % 18	84.6 % 22	52.0 % 7	50.0 % 6	67.9 % 53
Vahlkampfiidae	% Number	84.6 % 22	42.3 % 11	35.7 % 5	–	48.7 % 38
*Vermamoeba*	% Number	11.5 % 3	3.8 % 1	7.1 % 1	–	6.4 % 5
*Acanthamoeba* + Vahlkampfiidae	% Number	65.4 % 17	42.3 % 11	14.3 % 2	–	38.5 % 30
*Acanthamoeba* + Vahlkampfiidae + *Vermamoeba*	% Number	7.7 % 2	3.8 % 1	–	–	3.8 % 3
Total	Number	88.5 % (23/26)	84.6 % (22/26)	71.4 % (10/14)	50.0 % (6/12)	78.2 % (61/78)

^a^Relative frequencies of amoeba-positive samples in different sampling sites

^b^Absolute numbers of positive samples

Altogether, CT-Hospital 1 showed the highest frequency of occurrence of FLA, with 89 % positive samples (Table 1). This cooling tower also showed the highest frequency of occurrence of Vahlkampfiidae (84.6 %) and of *V. vermiformis* (11.5 %). There was no correlation between the frequency of occurrence of amoebae and the mode of disinfection, neither concerning the time schedule nor the disinfectants used.

### Isolation of FLA and identification of their endosymbionts

The tap water samples, including the sample from the shower head, were all negative for FLA by culture. Of the cooling water samples, 31 were positive for FLA in the initial cultures and 26 of these could be identified down to the genus level by morphologic characters. We aimed to obtain clonal monoxenic subcultures from all isolates of FLA; however, several cultures were lost due to fungal overgrowth despite treatment with anti-mycotics. Waters collected from CT-Hospital 1 also contained high numbers of mites and nematodes. Altogether, 16 isolates were successfully brought into monoxenic cultures and subjected to molecular analysis (Table 2). Nine different taxa could be identified by DNA sequencing. CT-Hospital 1 showed the highest amoebal diversity with four different genera (Table 2). Two isolates from CT-Hospital 2 were identified as *Naegleria* spp., with 100 % sequence homology to one another and with equal sequence identities to *N. clarki* and *N. pagei*. Both were shown to grow at 37 °C.

**Table 2:** Diversity of microorganisms per sampling site. FLA and *Legionella* isolated by culture and identified by DNA sequencing. Endosymbionts detected by FISH in isolates of FLA and identified by DNA sequencing

	FLA		Legionella	
Taxa	No. of isolates	Endosymbionts	Species	No. of isolates
Cooling tower Hospital 1				
*Acanthamoeba* genotype T4	3	*Paracaedibacter acanthamoebae*	*L. rubrilucens*	4
* Cochliopodium minus*	3	*Legionellales*		
* Stenamoeba* sp.	2	–		
* Thecamoeba sp.*	5	–		
* Protostelium*-like amoeba	1	–		
* Vahlkampfia avara*	1	–		
Cooling tower Hospital 2				
* Acanthamoeba* genotype T4	5	*Rickettsiales*		
Vahlkampfiidae^a^	1	*–*		
*Naegleria* clarki/pagei^b^	2	*–*		
Cooling tower Company				
*Acanthamoeba* genotype T4	1	–	*L. pneumophila* SG 2-14	1
*Leptomyxa reticulata*	2	–		
Shower head				
–	–	–	*L. pneumophila* SG 2-14	1
Total	26	3	6	

^a^Mixed culture of several genera grown at 30°

^b^Thermophilic

Three amoebal isolates, all from cooling towers, revealed endosymbionts (Table 2). In an *Acanthamoeba* isolate from CT-Hospital 1, the facultative intracellular bacterium *Paracaedibacter acanthamoebae* (order Rickettsiales) was detected. A *Cochliopodium minus* isolate from CT-Hospital 1 contained bacteria belonging to a new genus within the order Legionellales, distinct from the genus *Legionella*, and an *Acanthamoeba* isolate from CT-Hospital 2 contained a novel member of the order Rickettsiales (Tsao et al., in preparation).

### Routine screening for bacteria

Due to the extremely high organic burden in the water samples, 7 of the 66 cooling water samples had to be totally excluded. Despite acid treatment, for further 39 samples (39/59; 66.1 %) only smaller volumes, i.e. 1–10 ml, could be processed for routine screening. Thus, the standard volume of 100 ml could be analysed only from 33.9 % (20/59) of the cooling tower samples. Of these, 25 % (5/20) were positive for *Legionella* spp. From the five positive samples, four samples from CT-Hospital 1 were positive for *L. rubrilucens* in increased (>100–1000 CFU/100 ml) to highly increased (>1000 CFU/100 ml) concentrations and one sample from CT-Company was positive for *L. pneumophila* (serogroup 2–14) in low concentration (≤100 CFU/100 ml). The sample taken from the previously *Legionella*-positive hospital shower head also showed low concentrations (6 CFU/100 ml) of *L. pneumophila* serogroup 2-14 (Table 2). All other tap water samples investigated were negative. Also, all smaller volumes of water samples investigated remained negative in routine screening.

Of the 28 cooling tower samples analysed by amplicon sequencing, 14 (50 %) were positive for at least one *Legionella* OTU. While CT-Hospital 1 revealed three samples positive for *Legionella* spp. (21.4 %), from CT-Hospital 2 eleven (78.6 %) samples were positive. Each hospital cooling tower showed a unique set of *Legionella* OTUs, with no shared OTUs between the two. In total, we detected six different OTUs within the genus *Legionella*.

While all tap water samples investigated were negative, 25 % of the cooling tower waters were positive for *P. aeruginosa*. Eleven of these 16 samples showed low bacterial counts (1–100 CFU/100 ml) and 5 samples showed increased concentrations (>100–1000 CFU/100 ml). All samples with increased concentrations were from hospital cooling towers. Altogether, 56.3 % (9/16) of the *P. aeruginosa*-positive samples also were positive for FLA.

Concerning total heterotrophic bacteria, 56.3 % of the samples revealed low bacterial counts (1–10,000 CFU/ml), while increased numbers (>10,000–100,000 CFU/ml) were detected in 34.4 % of the samples and 9.4 % showed highly increased concentrations (>100,000 CFU/ml), all of them from hospital cooling towers. The tap water samples generally showed low concentrations of total bacteria.

## Discussion

With our new screening system for FLA, based on the combination of three group-specific real-time PCRs, we achieved a synchronous, rapid and highly sensitive detection of those amoebae that are most important as hosts for bacteria in water systems under permanent disinfection. It was shown that particularly the waters of hospital cooling towers have a high frequency of occurrence of FLA, even shortly after disinfection and despite having particularly stringent disinfection protocols. Moreover, it was shown that routine screenings for *Legionella* spp. do not give reliable results for waters with high organic burden.

With the real-time PCR-based screening system for FLA, we achieved a significantly higher sensitivity compared to a previous screening of industrial waters using culture and conventional PCR (Scheikl et al. 2014). As shown previously, culture had a low sensitivity, only 52.7 % of the real-time PCR positive samples being positive; however, the advantage of culture is that basically any viable species of FLA can be found and that isolated amoebae can be screened for intracellular bacteria.

Altogether, 83.3 % of the cooling tower water samples were positive for amoebae suited as hosts for bacterial survival and replication, despite regular disinfection and partly already 1.5 h after disinfection, independently of the disinfectants used. Interestingly, the two hospital cooling towers had a higher frequency of occurrence of FLA compared to the company cooling tower and, against expectations, the cooling tower from the hospital that had been the source of an *Legionella* outbreak in 2007 and since then had been dismantled and renewed, showed the highest frequency of occurrence of FLA. This cooling tower also had the highest organic burden and also the highest number of different amoebal taxa. At eight time points, disinfection had been performed shortly (1.5–3.5 h) before the sampling. All these samples still were positive for FLA, even in culture, demonstrating that disinfection had no effect on amoebal viability. From these cultures, among others, also one strain of *C. minus* was isolated that carried bacteria belonging to the Legionellales. Moreover, the co-occurrence of *C. minus* and *Legionella rubrilucens* was demonstrated in this hospital, CT-Hospital 1. FLA are a natural reservoir for legionellae and particularly open wet cooling towers provide optimal conditions for growth and dissemination of both of these taxa. *Acanthamoeba* and also *Cochliopodium* are known to be even stimulated by biocides recommended for cooling towers (Srikanth and Berk 1993). This once more corroborates the known reservoir function of amoebae for potentially pathogenic bacteria (Greub and Raoult 2004). Surprisingly, also vahlkampfiid amoebae were detected in all cooling towers, demonstrating that disinfection did not have a significant impact on even these more sensitive FLA compared to *Acanthamoeba* spp. (Pagnier et al. 2009; Canals et al. 2015).

*Acanthamoeba* showed the highest overall frequency of occurrence followed by vahlkampfiid amoebae, a majority of samples being positive for both. Atlan et al. (2012) revealed either *Acanthamoeba* or *Vermamoeba* as dominating genera in cooling towers and a predominance of *Acanthamoeba* was reported from water treatment plants (Magnet et al. 2013), while *V. vermiformis* was found to be the most abundant FLA species in drinking water (Thomas et al. 2008; Wang et al. 2012; Delafont et al. 2013; Pagnier et al. 2015), and in hot water systems (Rohr et al. 1998). In the current study, *V. vermiformis* was absent in tap water and also rarely found in cooling towers (7.6 %). This might be attributed to *Vermamoeba*’s higher sensitivity to biocides compared to *Acanthamoeba* (Coulon et al. 2010; Fouque et al. 2015). The higher frequency of occurrence of vahlkampfiids compared to a previous study on industrial waters (Scheikl et al. 2014) may be attributed to the higher sensitivity of the real-time PCR, but probably also to the warmer water temperatures. Vahlkampfiids are generally known to occur in cooling towers (Rohr et al. 1998; Canals et al. 2015). In the current study, also two thermophilic *Naegleria* strains, related to *N. clarki* and *N. pagei*, were isolated. Although both species are not considered pathogenic, they belong to the same phylogenetic cluster as *N. australiensis* and *N. italica*, which were shown to be pathogenic in animal experiments (De Jonckheere 2014).

Cooling tower waters with their constantly warm temperatures and a weak alkaline pH provide ideal conditions for *Legionella* spp., including *L. pneumophila* (Wadowsky et al. 1985), and also for FLA, including amoebae carrying bacteria (Berk et al. 2006; Huang et al. 2011; Buse et al. 2012). All *Legionella*-positive samples showed elevated temperatures, i.e. between 26 and 30 °C. The tap waters with temperatures <12 °C were all negative for *Legionella* spp., but the sample from the shower head was positive for *L. pneumophila* serogroup 2-14, although it had been cleaned and disinfected before. Three cooling tower water samples showed highly increased concentrations of *L. rubrilucens* (18,000–22,000 CFU/100 ml) and one sample increased numbers of *L. rubrilucens* (600 CFU/100 ml) although disinfected 1 day before sampling. *L. rubrilucens* always co-occurred with *Acanthamoeba* or Vahlkampfiidae or both amoebal groups.

Finally, our study indicates that the standard protocol for *Legionella* routine screening is not suitable for waters from open cooling towers with a high organic burden. For the majority of these samples, only reduced volumes can be investigated, resulting in unreliably low recovery rates for *Legionella*. In our study, only 7.6 % of all samples were positive for *Legionella* by culture. In comparison, the amplicon-based screening revealed the presence of *Legionella* spp. in 50 % of the samples investigated. In fact, the ISO 11731-2 (2004) standard protocol was initially established for waters with low bacterial counts (e.g. tap water), it is however, widely used for *Legionella* detection also from engineered waters. Non-standard molecular biological methods like CARD-FISH reveal higher numbers of *Legionella*-positive samples and also higher concentrations compared to the standard method (Kirschner et al. 2012). Co-culture with amoebae (Magnet et al. 2015) is another sensitive tool to isolate *Legionella* and other potentially pathogenic bacteria like chlamydiae, *Pseudomonas* spp. or mycobacteria from various sampling sources (Collingro et al. 2005; Thomas et al. 2008; Corsaro et al. 2010; Kebbi-Beghdadi and Greub 2014). However, both techniques have not yet been implicated into routine screenings. In the current study, *P. aeruginosa* was generally detected in low concentrations; increased concentrations were only measured at elevated temperatures (27 °C). Increased counts of *P. aeruginosa* and highly increased counts of total bacteria, respectively, were only detected in the hospital cooling towers. The majority of *P. aeruginosa*-positive samples was also positive for FLA, whereby *Acanthamoeba* always co-occurred with *P. aeruginosa*. Interestingly, all culture-positive samples for *Legionella* were negative for *P. aeruginosa*. It has been assumed that *P. aeruginosa* inhibits the growth of *L. pneumophila* (Kimura et al. 2009).

Altogether, our study highlights the need for a regular and regulated screening of cooling towers and for standardized modified screening protocols for these types of waters, possibly also including FLA.

## Conclusions

In conclusion, the newly established real-time PCR-based screening system for amoebae is well-suited for a regular and synchronous screening for various groups of FLA in water samples. It was demonstrated that there is a high frequency of occurrence of amoebae suited as hosts for bacteria in cooling towers, particularly in hospital cooling towers, and that regular disinfection does not affect amoebal survival. Moreover, it was shown that results obtained from *Legionella* routine screenings are not always reliable for water samples with high organic burden.
